# Of Medical Certificates

**Published:** 1918-03-02

**Authors:** 


### OF MEDICAL CERTIFICATES.

There are many varieties of medical certificates used for various purposes. Some are required by the law, and are bare statements of fact, like death certificates; others are more ample and are paid for at no exorbitant rate by County or Borough Councils; others, again, are given at the will of the doctor and the request of the patient; whilst yet others are obtained by the recipient from the doctor, who is a more or less willing agent. It is of these latter certificates that speech is to be made at the present time. They may be divided into two great groups—those which are founded on fact and state facts, like the great majority of certificates given in regard to actual physical defects; and those constructed with the intention to mislead, like many certificates dealing with obscure symptoms, and certificates based upon a minimum of signs and an exaggeration of symptoms. These latter certificates are not to be obtained of all medical practitioners, and when they are obtained, their cost is out of all proportion to the value attached to them when they come under the consideration of those who are accustomed to appraise them. They bear such a strong family resemblance to each other as betrays their nature, and often their very source, to the experienced. Some are couched in recondite terms—sonorous, and full of mystery to the recipient, mere tinkling brass to those they are intended to influence, for the very expressions used are indications of the poverty of fact upon which they are based. Others obtrude palpable defects which have been borne patiently for years, so long as the patient was carrying on civil duties, but which suddenly develop into ills too great for human endurance when the man-power of the nation is under revision. Indigestion then becomes acute gastric catarrh; every fit, even a fit of temper, is liable to be called epilepsy; a cough is a certain precursor of tuberculosis, flat feet and hammer-toes become veritable bugbears, and cause the patient to hobble badly even when his soles are free from callosities, and his toes from corns. The effect of such certificates seems to be as disastrous to the doctor as to the patient. Given at first from mere indolence and out of good-nature, it soon becomes a habit. The more certificates he gives, the more he may give, for he becomes surrounded and engulphed by a crowd of applicants each desiring to obtain what he is only too willing to give, neither party counting the cost. *Facilis decensus Averni*; it is easier to sit at home and write certificates than to go out and see the sick poor. Very soon the poor are neglected altogether, and the pile of certificates increases until the doctor loses his self-respect and will state palpable untruths. There remains a last class of certificate, which is the rarest but not the least interesting. It is the forged certificate, usually written by the patient himself or by a friend, signed with the name of a doctor whom he has picked out at random. Such certificates are as easy to detect as Bank of Engraving notes. The perpetrators are usually illiterate, and if by chance they get the name of the disease correctly, they are hopelessly at sea about the qualifications to be appended to the doctor's name.

(*Continued on page* 458.)

**Of Medical Certificates** (*cont. from p.* 456.)

The general outcome of these considerations is that every certificate should be carefully weighed. The reasons for which it is required should be considered, and neither good-nature nor a greed for pelf should induce anyone to exaggerate slight symptoms or make diagnoses which are designedly false. As a deterrent, it should be borne in mind that, all medical certificates eventually come into the hands and under the careful scrutiny of a few experts, with long memories.

